# Sexual Violence toward Children and Youth in War-Torn Eastern Democratic Republic of Congo

**DOI:** 10.1371/journal.pone.0015911

**Published:** 2011-01-18

**Authors:** Luc Malemo Kalisya, Paluku Lussy Justin, Christophe Kimona, Kavira Nyavandu, Kamabu Mukekulu Eugenie, Kasereka Muhindo Lusi Jonathan, Kasereka Masumbuko Claude, Michael Hawkes

**Affiliations:** 1 HEAL Africa, Goma, Democratic Republic of Congo; 2 Institute of Medical Sciences, University of Toronto, Toronto, Ontario, Canada; The University of Queensland, Australia

## Abstract

**Background:**

The epidemic of gender-based violence in the Democratic Republic of the Congo (DRC) has garnered popular media attention, but is incompletely described in the medical literature to date. In particular, the relative importance of militarized compared to civilian rape and the impact on vulnerable populations merits further study. We describe a retrospective case series of sexual abuse among children and youth in eastern DRC.

**Methods:**

Medical records of patients treated for sexual assault at HEAL Africa Hospital, Goma, DRC between 2006 and 2008 were reviewed. Information extracted from the chart record was summarized using descriptive statistics, with comparative statistics to examine differences between pediatric (≤18 yrs) and adult patients.

**Findings:**

440 pediatric and 54 adult sexual abuse cases were identified. Children and youth were more often assaulted by someone known to the family (74% vs 30%, OR 6.7 [95%CI 3.6–12], p<0.001), and less frequently by military personnel (13% vs 48%, OR 0.14 [95%CI 0.075–0.26], p<0.001). Delayed presentation for medical care (>72 hours after the assault) was more common in pediatric patients (53% vs 33%, OR 2.2 [95%CI 1.2–4.0], p = 0.007). Physical signs of sexual abuse, including lesions of the posterior fourchette, hymeneal tears, and anal lesions, were more commonly observed in children and youth (84% vs 69%, OR 2.3 [95%CI 1.3–4.4], p = 0.006). Nine (2.9%) pediatrics patients were HIV-positive at presentation, compared to 5.3% of adults (p = 0.34).

**Interpretation:**

World media attention has focused on violent rape as a weapon of war in the DRC. Our data highlight some neglected but important and distinct aspects of the ongoing epidemic of sexual violence: sexual abuse of children and youth.

## Introduction

Violent conflict in the Democratic Republic of the Congo (DRC) over the past decade has been associated with a large-scale epidemic of rape [1], [2]. The alarming brutality and scale of sexual violence in some areas of eastern DRC has resulted in thousands of cases of traumatic genital injury and gynecologic fistulae among adult female victims, together with devastating psychological and social consequences [1], [2], [3], [4].

Much attention has focused on military personnel using sexual violence as a deliberate tactic to terrorize, displace, and demoralize local communities [1]. Some cases of rape reportedly occur in full, deliberate and enforced view of husbands and family members [1]. Indeed, rape has been used throughout history as a weapon of war [5], most memorably in recent conflicts in Bosnia [6] and Rwanda [5]. Sexual violence committed during these conflicts has been recognized as systematic and politically-motivated: sexual assault was declared a crime against humanity by International Tribunal for the Former Yugoslavia (1996), and an act of genocide by the International Tribunal for Rwanda (1998) [5].

On the other hand, with the breakdown of social structures during times of war, sexual exploitation of vulnerable groups may occur without political motivation. The situation may be exacerbated by weakened judicial and law enforcement systems leading to a climate of impunity, or possibly a “normalization” of rape among the general population in times of violent conflict [7]. Following a complex civil war and international conflagration that has claimed over 5 million lives [8], [9], multiple armed militias remain in eastern DRC. They lack a central command structure, and are responsible for numerous atrocities, looting and destruction [2]. Sexual crimes by renegade soldiers in the area may not represent a concerted political effort as much as random acts of violence by assailants in positions of power over their victims. Similarly, it has been suggested during the Rwandan conflict that for many perpetrators, rape was another form of delinquency, like theft or looting [5]. Furthermore, a significant proportion of sexual assaults in the DRC involve non-military perpetrators, such as the police and civilians [2].

Here we describe a large cohort of pediatric sexual assault victims seen at HEAL Africa Hospital in Goma, DRC over a three year period from 2006 to 2008. Our findings highlight the predominance of domestic sexual abuse over militarized rape in this vulnerable population, which has been relatively neglected in previous descriptions of the epidemic of sexual violence in the DRC.

## Methods

### Ethics statement

The medical director and executive director of HEAL Africa hospital provided ethics approval for this study. Data were anonymised and de-identified at the point of data abstraction from the chart record. Thereafter, for the purposes of analysis and write-up, patient data could not be linked to any identifying personal health information.

### Setting

Over the past decade, the DRC has been the stage of one of the most deadly humanitarian crises in recent history [8], [9]. Despite an official end to the conflict, mortality rates remain 70% higher than pre-war levels and 55% higher than surrounding sub-Saharan African countries [8]. Gender-based violence continues to plague the eastern regions of the DRC, with up to 5.0% of households reporting at least one episode of rape in population-based surveys in some areas of Eastern DRC [10]. Access to health care in the area is good and often free of charge, in part due to services provided by international non-governmental organizations [11]. In this setting, HEAL Africa hospital has provided medical services to the city of Goma and surrounding areas throughout the turbulent events of the past decade. In addition to a large surgical service dedicated to the repair of gynecologic fistulae, HEAL Africa also has numerous community outreach projects, including *Guéris Mon Peuple*, an initiative to combat gender-based violence. It is recognized as a pre-eminent medical hospital in the region and thus serves as a referral centre for women and children who are victims of sexual assault from Goma and its surroundings. HEAL Africa's activities in a zone of violent conflict provide evidence of the types of positive contributions that can be made in practice by the health sector to promote “peace through health [12].”

### Patients

Medical records of all children and adults treated at HEAL Africa Hospital for sexual violence from 2006 to 2008 were reviewed. The information extracted from the chart record included sex, age of victim, details on the perpetrator (provided by the victim's caregiver), findings from the initial physical examination, testing, and treatment provided.

### Statistical analysis

Summary statistics were calculated separately for children and youth (18 years of age and under) and adults. Comparative statistics were computed using SPSS 17.0. Where multiple potential confounding variables were present, we used multivariate logistic regression models to determine independent predictors of important outcomes (e.g., delayed presentation).

## Results

A total of 440 cases of pediatric sexual abuse were identified. In addition, we identified 54 adult sexual abuse victims over the same time period. Despite an official end to armed conflict in the area in 2003, the annual number of pediatric rape cases presenting for medical care (129, 162, 149) and the proportion of pediatric rape allegedly perpetrated by military personnel (14%, 16%, 22%, p-value for trend = 0.072) did not decrease over the years of the study (2006, 2007, 2008, respectively).

Table 1 shows characteristics of pediatric rape victims, with comparative data from adult victims seen at HEAL Africa hospital over the same time period. Nearly all pediatric patients (98%) were female. Compared to adult patients (n = 54), patients below 18 years of age (n = 440) were more often assaulted by someone known to the family (74% vs 30%, OR 6.7 [95%CI 3.6–12], p<0.001), and less frequently by men in military uniform (13% vs 48%, OR 0.14 [95%CI 0.075–0.26], p<0.001). Of note, it is difficult to know if uniformed assailants were affiliated with any organized military group, or represented civilians in military dress. Daytime sexual assault was more common in children and youth (66% vs 48%, OR 2.1 [95%CI 1.2–3.7], p = 0.009).

**Table 1:** Features of pediatric sexual assault cases compared to adult cases seen in Goma, eastern DRC.

Factor	Pediatric victims (n = 440)	Adult victims (n = 54)	p-value
**Yearly distribution**			
2006	129	11	0.003
2007	162	12	
2008	149	31	
**Age of victims ( years)**			
<5	60 (14)		
6–10	50 (11)		
11–18	330 (75)		
>18		54	
**Sex of victims**			
Female	433 (98)	52 (96)	0.26
Male	7 (1.6)	2 (3.7)	
**Time of assault**			
Day	291 (66)	26 (48)	0.009
Night	149 (34)	28 (52)	
**Description of assailant**			
Civilian	358 (81)	23 (43)	<0.001
In military uniform*	57 (13)	26 (48)	
Unknown	25 (5.7)	5 (9.3)	
**Relationship with assailant**			
Known to family	322 (74)	16 (30)	<0.001
Stranger	114 (26)	38 (70)	
**Time to consultation**			
<72h	208 (47)	36 (67)	0.007
>72h–1 week	51 (12)	2 (3.7)	
>1 week	181 (41)	16 (30)	
**Signs found at examination**			
Hymeneal tear	254 (58)	21 (39)	0.006
Vulvar lesions	89 (20)	13 (24)	
Anal lesions	6 (1.4)	0	
Other lesions	19 (4.3)	3 (5.6)	
No lesion	72 (16)	17 (32)	
**Pregnancy, positive/number tested (%)** ^1^	85/407 (21)	4/52 (7.7)	0.024
**Sexually transmitted infections**			
HIV test, positive/number tested (%)	9/307 (2.9)	2/38 (5.3)	0.34
HIV post-exposure prophylaxis, given/eligible (%)^2^	129/193 (67)	31/35 (89)	0.009
VDRL test at presentation	14/308 (4.6)	1/37 (2.7)	0.93

*In eastern DRC, the presence of multiple armed groups without central leadership, deserters known as “inciviques [11],” widely available military uniforms in the post-war period, and the possibility that civilians may wear military uniforms leaves open the possibility that patients' description of uniformed assailant may not always be military personnel.

**Figure 1:**
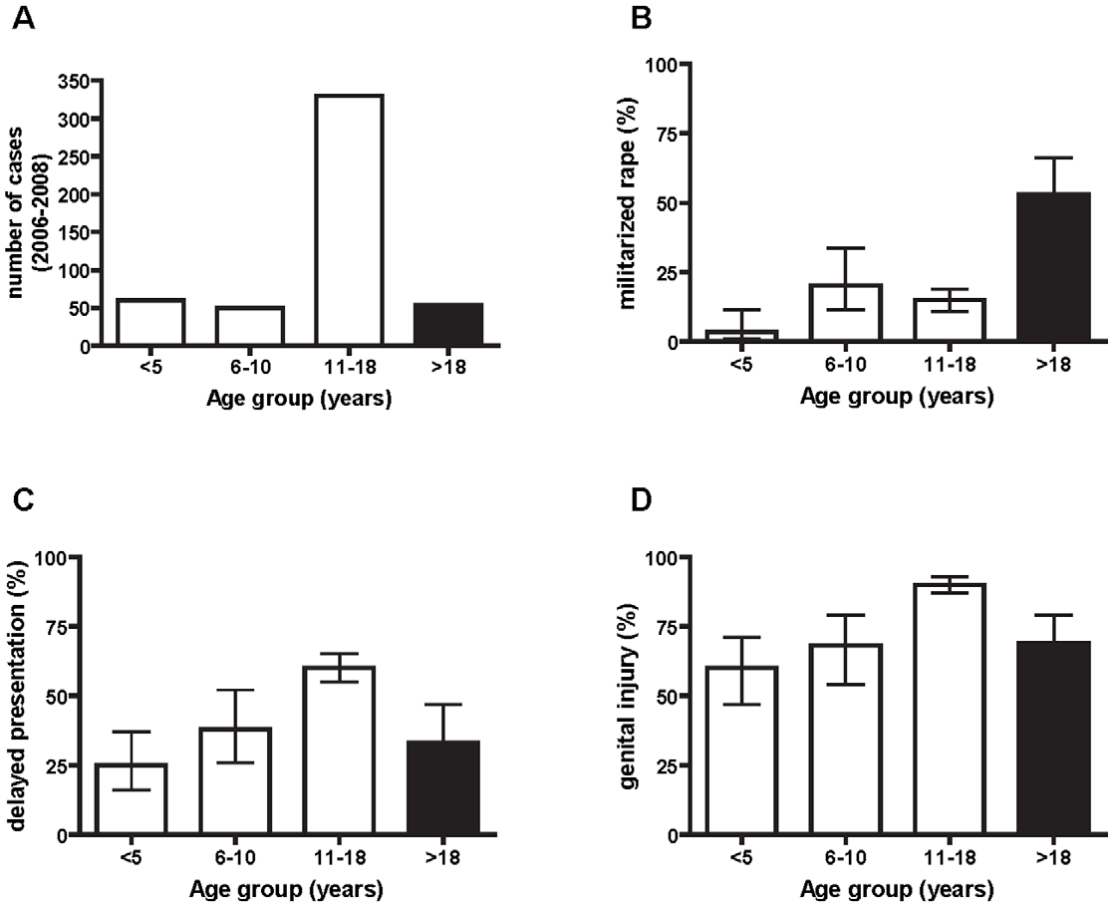
Characteristics of sexual assault according to patient age. **A**.The largest number of cases in our retrospective sample from a single centre between 2006 and 2009 was in the age group 10–18 years. **B**. The proportion of cases allegedly perpetrated by military personnel was significantly higher in adult compared to pediatric rape victims (p<0.001). **C**. Delayed (>72hours) presentation was more common in youth aged 10–18 years than adults and younger children. **D**. Genital injury was more commonly observed on physical examination at presentation in youth aged 10–18 years than adults and younger children.

Fifty-three percent of pediatric rape victims presented for medical care more than 72 hours after the assault, compared to 33% of adults (OR 2.2 [95%CI 1.2–4.0], p = 0.007). Predictors of delayed presentation in our pediatric cohort included age category and daytime assault, whereas the identity of the perpetrator was not a significant predictor (Figure 1). Age and timing of assault remained significant independent predictors of delayed presentation in a multivariate logistic regression model adjusting for possible confounding effects. Of note, delayed presentation was most common in adolescent patients (age 10–18) who experience daytime rape, whereas young children and adults tended to present for care earlier (Figure 1).

Physical signs of sexual abuse, including lesions of the posterior fourchette, hymeneal tears, and anal lesions, were more commonly observed in children and youth than adult rape victims (84% vs 69%, OR 2.3 [95%CI 1.3–4.4], p = 0.006). Patient age was the only statistically significant predictor of genital trauma in univariate analysis; the identity of the assailant (known to the family vs unknown, military vs civilian), timing of the incident, and delayed presentation were not (Figure 1).

Testing for pregnancy was performed in 407 (93%) of pediatric patients and was positive in 85 (21%) cases, compared to 7.7% of adults (OR 3.2 [95%CI 1.1–9.0], p = 0.024). HIV testing was performed and documented in 307 (70%) of pediatric patients and in 38 (70%) of adult patients at presentation (p = 0.93). Nine (2.9%) pediatrics patients tested positive at presentation, compared to 5.3% of adults (p = 0.34). Pediatric patients presenting within 72 hours of sexual assault received post-exposure prophylaxis with combined zidovudine and lamivudine in 67% of cases, compared to 89% of adults (OR 0.26 [95%CI 0.088–0.77], p = 0.009). VDRL testing was positive in 14 of 303 pediatric patients tested (4.6%), compared to 2.6% of adults tested.

## Discussion

This case series of sexual abuse victims seen at a referral hospital in war-torn eastern DRC highlights distinct features of pediatric relative to adult rape in the area. In particular, domestic abuse predominates over militarized rape in this age group. Novel aspects of this study include its focus on children and youth, who have been largely under-studied in other reports of sexual abuse from the area. To our knowledge, this represents the largest case series of pediatric sexual assault from Africa to date.

Our patient sample consists of a hospital-based cohort presenting for treatment for alleged sexual violence. Other hospitals in the area have also reported large case series of gender-based violence, supporting our finding that the epidemic of rape persists in the area [1], [4]. Population-based studies likewise demonstrate high levels of violence in the area, with up to 5% of households in some communities in Eastern DRC reporting at least one episode of rape, although rates vary widely across the region [10], [11]. Access to health care in the area is good, at least in selected communities served by international non-governmental organizations: according to one recent survey, 80–93% of the sick received care at a health facility, of whom 83–90% received these services without fee [11]. This suggests that many rape victims may access hospital services for their care, although additional barriers such as stigma and shame likely affect health seeking behavior. Thus, it is likely that our hospital-based sample represents only a small fraction of sexual abuse cases in the community, with possible over-representation of more severe cases requiring medical attention.

Pediatric patients comprised a significant proportion of reported rape victims seen at HEAL Africa hospital. At the Panzi Hospital in the neighboring province of South Kivu, approximately 10% of reported rape victims were under 15 years of age, apparently a smaller proportion of children than was observed in our study. Because theirs was a non-systematic convenience sample of women selected for interview based on the perceived severity of physical or psychological trauma, children may be under-represented [4]. In contrast, our study included all patients seen at the hospital between 2006 and 2008 for sexual violence. The scant population-based studies in the region that describe sexual violence in the area have not reported on the proportion of child victims [10], [11]; therefore, it is unclear to what extent our hospital-based cohort is representative of rape victims in the community at large.

The majority of perpetrators of child sexual abuse were described as civilians (81%) and known to the family (74%). Nonetheless, militarized rape among children and youth in our series shared similar features of cruelty and extreme violence previously described in adults [1]. We observed no change over time in the number of cases or the proportion of rape perpetrated by military personnel. In comparison, a recent report carried out by the Harvard Humanitarian Initiative revealed disturbing rape trends in Eastern Congo. The study found that although reported assaults decreased between 2004 and 2008, there has been a 17-fold increase in the number of reported civilian rapes. These findings may imply a normalization of rape among civilians and indicate that current mechanisms meant to protect women from violence are ineffective [7]. Alternatively, increasing health-seeking behavior, access to care, and/or willingness to report to medical professionals may account for the higher number of civilian sexual assault cases observed over time.

The majority (75%) of pediatric victims were between the age of 10 and 18 years. Over 95% of pediatric and adult patients were female, which may be attributable, at least in part, to under-reporting of sexual abuse of males. The age and sex distribution was similar to previous reports from several African countries [13], [14], [15], [16], [17]. Delayed presentation, pregnancy and genital injury were more common in this age group than adults and younger children (Figure 1). Possible explanations for this finding include: dependence of youth and adolescents on a caregiver to present to the hospital; shame or stigma delaying disclosure in this age group; or deliberate interference with timely medical attention by an influential assailant. The majority of children and youth in our study (74%) were acquainted with the rapist. Similar observations have been reported in Lesotho [18], Uganda [15], Brazzaville Congo [17], and Kenya [13]. Thus, patterns of sexual violence among children and youth in the DRC resemble those in many other African states.

In the majority of cases, there was evidence of penetration. These findings are similar to reports from Australia [19], Brazzaville Congo [17], and Uganda [15]. However, a normal physical examination does not rule out rape [16], [19], [20], [21]. Patient age, but not the identity of the assailant, was a predictor of the presence of genital injury in our study, in contrast to another report where unknown assailant was associated with increased risk of genital trauma [19].

Prompt presentation for medical care is crucial for the timely administration of post-exposure prophylaxis for HIV, treatment of other STIs, and management of pregnancy risk. In our pediatric cohort, approximately half of patients presented 72 hours or more after the assault, and delayed presentation was more common in patients 10–18 years old. Similarly, 76% of patients presented beyond 72 hours in another study [21]. Some reasons for delayed presentation in our area may include lack of awareness of ideal timing for STI prophylaxis, financial constraints, geographic barriers, and shame associated with disclosure of sexual violence. In one study, 100% of 153 women arrived at the hospital within 72 hours of their assault, suggesting that education campaigns for young girls might address remediable barriers to early presentation in our setting. Furthermore, our finding that 67% of eligible pediatric patients received post-exposure prophylaxis for HIV suggests that opportunities for improved management exist in the pediatric age group.

Limitations of this study include data from a single centre which may affect the generalizability of our findings. The retrospective, observational nature of the study restricts our ability to examine causal relationships between variables. This study examined medically-attended sexual assault cases, which likely represent only a small fraction of the total cases in the community at large. Case ascertainment from retrospective chart review may not have been complete over the study period. Low rates of STIs in our sample prevented us from examining predictors of STI among rape victims. Loss-to-follow-up prevented us from examining seroconversion rates for HIV and VDRL.

In conclusion, the profile of pediatric sexual abuse in eastern DRC is distinct from that of adults. Whereas world media attention has focused on militarized rape used as a weapon of terror, our data underscore an important but neglected aspect of the epidemic of sexual violence in the Congo: domestic sexual violence directed against children and youth.
